# Investigating the validity and reliability of Electrovestibulography (EVestG) for detecting post-concussion syndrome (PCS) with and without comorbid depression

**DOI:** 10.1038/s41598-018-32808-1

**Published:** 2018-09-27

**Authors:** Abdelbaset Suleiman, Brian Lithgow, Behzad Mansouri, Zahra Moussavi

**Affiliations:** 1 Biomedical Engineering Program, University of Manitoba, Winnipeg, MB Canada; 2 Department of Internal Medicine (Neurology), University of Manitoba, Winnipeg, MB Canada; 3 Monash Alfred Psychiatry Research Center, Monash University, Melbourne, Australia

## Abstract

Features from Electrovestibulography (EVestG) recordings have been used to classify and measure the severity of both persistent post-concussion syndrome (PCS) and major depressive disorder. Herein, we examined the effect of comorbid depression on the detection of persistent PCS using EVestG. To validate our previously developed EVestG classifier for PCS detection, the classifier was tested with a new blind dataset (N = 21). The unbiased accuracy for identifying the new PCS from controls was found to be >90%. Next, the PCS group (N = 59) was divided into three subgroups: PCS with no-depression (n = 18), PCS with mild-depression (n = 27) and PCS with moderate/severe-depression (n = 14). When moderate/severe depression was present, PCS classification accuracy dropped to 83%. By adding an EVestG depression feature from a previous study, separation accuracy of each PCS subgroup from controls was >90%. A four and three-group (excluding mild-depression subgroup) classification, achieved an accuracy of 74% and 81%, respectively. Correlation analysis indicated a significant correlation (R = 0.67) between the depression feature and the MADRS depression score as well as between the PCS-specific feature and Rivermead Post-Concussion Questionnaire (RPQ) (R = −0.48). No significant correlation was found between the PCS-specific feature and the MADRS score (R = 0.20) or between RPQ and the depression feature (R = 0.12). The (PCS-specific and depression-specific) EVestG features used herein have the potential to robustly detect and monitor changes, relatively independently, in both persistent PCS and its depression comorbidity. Clinically, this can be particularly advantageous.

## Introduction

Individuals, who sustain a head injury, are usually affected by a cluster of cognitive, somatic and emotional symptoms for periods of time. These symptoms usually include headaches, dizziness, fatigue, irritability, reduced concentration, sleep disturbance, memory loss, sensitivity to noise or light, double or blurred vision, nausea, anxiety and depression^1,2^. These symptoms, except headache and dizziness, are often reported to last for a few days and up to a few weeks following the injury. When these symptoms persist more than a month or so, it is called persistent post-concussion syndrome (PCS). Why and how PCS develops and changes over time has remained controversial for decades. However, it is certain that persistent PCS occurs due to pathophysiological changes occurring after a mild traumatic brain injury (mTBI)^3^ (commonly called a concussion).

One of the most concerning symptoms or psychiatric diagnosis after a head injury is post-concussive depression. The estimated rate of depression in the first year following a mild to severe traumatic brain injury (TBI) ranges from 26 to 53%^4–7^. Many studies have shown an association between PCS severity and depression severity after mTBI^8,9^. Moreover, it was shown that the diagnosis of PCS might be overlooked in favour of a diagnosis of depression^10^.

There are several diagnostic tools for concussion diagnosis, which are used either alone or in combination. These include the neuropsychological assessments, such as the Rivermead Post-Concussion Questionnaire (RPQ)^11,12^; The Glasgow Coma Scale (GCS)^13^, the Immediate Post-Concussion Assessment and Cognitive Testing (ImPACT)^14^, the Sports Concussion Assessment Tool and the Sport Concussion Assessment Tool version 5 (SCAT5)^15^. However, it is not recommended to use these assessment outcomes as the sole basis for clinical decision making, as they may be biased by several confounding factors such as intelligence, age, education, depression and malingering^16^.

The most used objective techniques are neuroimaging techniques^15,17^, such as computed tomography (CT) or susceptibility weighted (SWI) magnetic resonance imaging (MRI)^18^. While these techniques have been shown to be sensitive for identifying moderate/severe TBI where lesions and structural fractures are more likely to exist^18,19^, they provide little contribution to PCS evaluation as the injury to the neural tissues is micro-structural and is usually not detected with imaging^15^. More advanced techniques such as positron emission tomography (PET), diffuse tensor imagining (DTI), have more positive outcomes^19^. However, these techniques still in the early stages of development and cannot be recommended other than in research settings^19^.

Quantitative electroencephalogram (qEEG)^20–22^ is another tool which has been used for PCS detection. It has shown a positive outcome in predicting the severity of head trauma and can also provide information on the long-term prognosis. The accuracy obtained for detecting PCS using qEEG is as high as 95.6% for short-term TBI^22^ and 77% for predicting the existence of the PCS one year after injury^23^. However, qEEG is not clinically used yet and still some studies question its clinical usefulness^24,25^.

**Figure 1:**
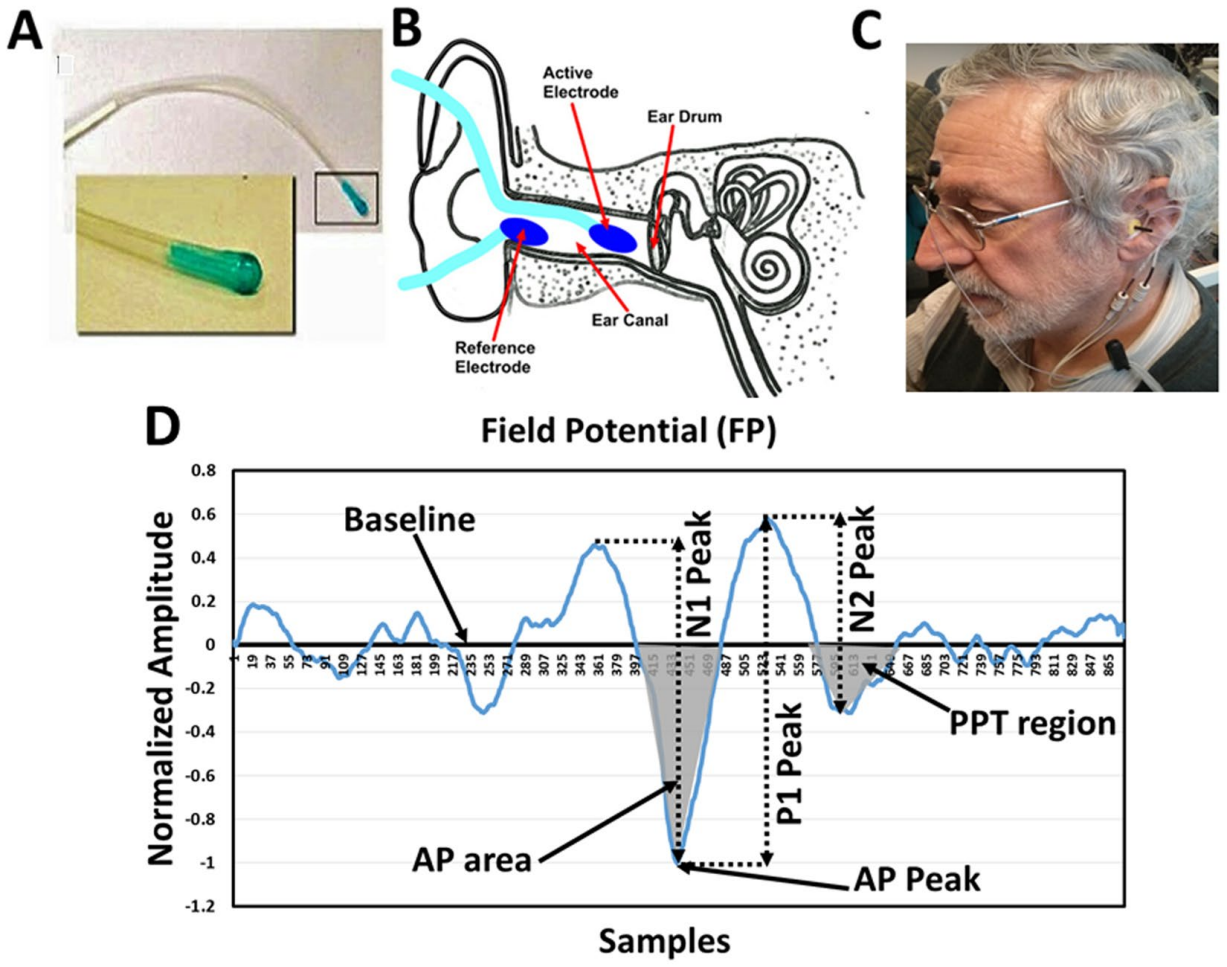
(**A**) Ear electrode; (**B**) electrodes placement; (**C**) participant connection (**D**) A typical normalized field potential (FP) (Horizontal scale 41.6 samples = 1 ms). AP-area: the bounded area between the baseline and the AP peak (marked area). PPT area: the bounded area between the samples (571:619) which represents the PPT range of control population.

Electrovestibulography (EVestG)^26–28^ that measures vestibulo-acoustic predominantly vestibular response changes^29–31^. The recorded signal is a combination of acoustic and vestibular generated field potentials (FPs)^32^. EVestG measures the predominantly vestibular response either statically or in response to passive whole body tilts from the external ear (Fig. 1). It is used for PCS diagnosis because vestibular deficiencies commonly occur after a head injury; these include dizziness^33,34^, imbalance and vertigo^35,36^. Up to 81% of the PCS population experienced dizziness within the first three months after the injury and 23% of the cases continued experiencing dizziness beyond 6 months^33,34^.

In a recent study^26^, two features of the average FPs of the EVestG were shown to identify PCS individuals from age-and-gender-matched healthy individuals with an 84% leave-one-out cross-validated test accuracy when compared to their clinical diagnosis. The two EVestG features were: (1) the area under the baseline and the action potential (AP) point of the average FP signal (Fig. 1D) called AP-area, and (2) relative to controls, the distribution wise change of the FP firing pattern (called IH33). The AP area feature predominantly measures the efflux and influx of sodium and potassium ions of the afferent vestibular nerve, while the IH33 feature is hypothesized to represent the spontaneous activity of the efferent nerve and or α band activity^26^.

An EVestG feature extracted from post potential trough (PPT) region of the average FP (Fig. 1D), is known to be sensitive to depression. It has been previously applied with two other Depression features as an aid to diagnose major depressive disorder with an ~87% accuracy^32^. It was similarly described in a depressive phase bipolar disorder study^37^. Given the common comorbidity of depression with PCS, it is necessary to question whether the PCS classifier is, at least partially, affected by the presence of depression and whether the EVestG PCS features used herein and in^26^ for PCS classification are correlated with the PPT region depression feature. These questions are addressed in this paper.

The goals of this study were two-fold: (1) is to test the validity of our previously developed EVestG classifier for PCS detection^26^ using a new blind dataset, and (2) to test whether the EVestG-PCS classifier is affected by co-morbid depression. Thus, we used the PCS classifier trained and cross-validated on 38 PCS and 33 healthy age-gender-matched controls data from our previous study^26^ and tested it on a newly recorded PCS dataset of 21 individuals. Next, we combined the two PCS datasets (N = 59) and grouped them based on their comorbid depression level based on their Montgomery–Åsberg Depression Rating Scale (MADRS) score. Three groups were formed: PCS with no current depression (MADRS ≤ 6, n = 18), PCS with mild depression (7 ≤ MADRS ≤ 19, n = 27), and PCS with moderate/severe depression (MADRS ≥ 20, n = 14). Then, we used the PPT feature (Fig. 1D) of EVestG shown to be sensitive to depression^32^ to help differentiate the above three PCS groups and investigate the correlation of the depression-specific feature with the two PCS specific features. We then determined the correlations of EVestG depression and PCS features (IH33 and AP-area) with the standard neuropsychological assessments used in this study as well as with each other as an indicator of their independence.

## Results

The inclusion/exclusion criteria for PCS group were: (1) being over 15 years of age, (2) having at least one head trauma with or without loss of consciousness in the last 10 years, (3) having a GCS scale >13 within 10 minutes after the head trauma, (4) having continued symptoms and signs of concussion one month after the head trauma at the time of neurological examination (e.g. blurred/double vision, vertigo, headache, imbalance, mood/cognitive/sleep abnormalities, convergence insufficiency, eye misalignment, cerebellar/vestibular abnormality, cognitive abnormality), and (5) having normal hearing. The healthy control group’s inclusion criteria were: (1) being over 15 years of age, (2) have no history of head trauma, ear infection/injury, any psychiatric and/or neurological disorder, and (3) having normal hearing.

All participants were referred from the neuro-ophthalmology clinic after being diagnosed with PCS and met the inclusion/exclusion criteria for the study. The diagnosis of PCS was conducted by the study neuro-ophthalmologist (author BM).

Table 1 shows demographic information and duration of the injury of the data adopted from the previous study and the new data recorded in this study, and Table 2 shows the demographic information of the PCS and depression subgroups. All data were recorded at the Neural Diagnostic Laboratory, Riverview Health Center, Winnipeg, Manitoba, Canada. All participants signed an informed consent approved by the Biomedical Research Ethics Board of University of Manitoba prior to recording.

**Table 1:** Demographics table of PCS when they were divided into subgroups based on their MADRS score (PCS with no depression, PCS & mild depression and PCS & moderate/severe depression.

Descriptive variables	PCS with no depression (n = 18)	PCS & mild depression (n = 27)	PCS & moderate/severe depression (n = 14)
Sex, female	11	17	10
Age (SD)	42.5 (15.8)	41.4 (13.9)	48.1 (10.2)
Time (years (SD))	0.8 (0.6)	2.1 (3.4)	3.5 (5.3)
MADRS (SD)	4.6 (1.6)	11.0 (3.5)	24.7 (2.6)
SPCS (n)	9	6	0
LPCS (n)	9	21	14

**Table 2:** Demographics table of PCS, LPCS, SPCS and healthy controls.

Descriptive variables	PCS (n = 59)	LPCS (n = 44)	SPCS (n = 15)	Control (n = 33)
Sex, female	38	30	8	20
Age (SD)	43.3 (12.9)	43.42 (12.48)	42.8 (15.6)	42.5 (16.2)
Time since injury (years (SD)) [Range]	2.2 (4.4)	2.9 (4.8) [5 months- 5 yrs]*	0.1 (0.09) [1 week – 3 months]	N/A
MADRS (SD)	12.6 (8.1)	14.3 (8.4)	7.7 (4.7)	4.1(1.3)

*4 participants were > 8 yrs.

Figure 1D shows an average FP plot, extracted from the EVestG signal using the wavelet-based signal processing technique called the Neural Event Extraction Routine (NEER)^29^, with areas marked locating where both the PCS and depression features are extracted. An average FP plot can be extracted by the NEER algorithm from all static (stationary—no movement) and dynamic (movement evoked) segments of an EVestG recording. In this current study, however, we analyzed only the static segments (no movement) to be congruent with our method in the previous study. In general, static segments have the least number of movement artefacts.

### EVestG PCS Features

**Figure 2:**
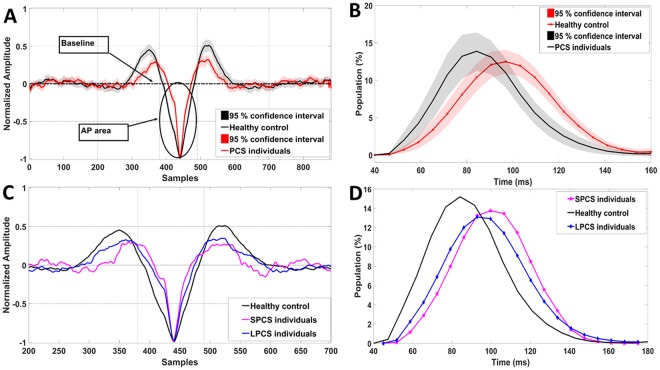
(**A**) Average response for control (n = 33) and PCS (long and short-term PCS, n = 59) groups. The marked circles/arrows show significant (P < 0.05) difference in the AP-area between control and concussed during static segment. (**B**) Interval Histogram for an FP gap equal to 33 FPs during static segment. The black and red solid lines represent the healthy controls (n = 33) and PCS (long and short-term PCS, n = 59) encased by dashed 95% confidence interval lines, respectively. (**C**) Average response for control (n = 33), SPCS (n = 15) and LPCS (n = 44), x-axis was zoomed-in to samples (200:700) to make the AP-area change more visible. (**D**) Interval Histogram for an FP gap equal to 33 FPs during static segment. The black, blue and pink solid lines represent the healthy controls (n = 33), SPCS (n = 15) and LPCS (n = 44) respectively.

*Action potential (AP) area* represents the area bounded between the baseline and the AP point (Fig. 1D) of the normalized FP. This feature was found significantly different between controls and PCS participants in previous studies^26,27^. It showed a high classification accuracy (feature ROC = 0.84) between controls and PCS groups. Similar to our previous studies, the AP area was extracted from the static segments; the average AP area was considered an EVestG PCS-specific feature. Figure 2A shows the average FP with marked AP-area for 33 healthy controls and all 59 PCS data.

Beside the FP curve, the NEER records the time of occurrence of each detected FP. The average experimentally measured time gap that NEER detects between two successive FPs is ~3.3 ms. Therefore, a 33 FP gap corresponds to about ~100 ms (10 Hz)^32^. *The IH33 feature is* a measure of the low frequency (~10 Hz) modulations of spontaneous (and driven) FP interval activity. It has been hypothesized the low-frequency activity occurs in response to efferent and or α band activity^32^. Spontaneous vestibular efferent activity is seen at 10–50 spikes/sec^38^, and the α band is 8–13 Hz. The average interval histogram based on the 33^rd^ (IH33) FP gap during the average static segments was then generated. This IH33 feature was also used in a previous PCS study^26^ and showed promise as a feature for separating controls and PCS (ROC = 82%). Figure 2B shows the IH33 histogram with 95% confidence intervals for the two groups of healthy controls and PCS dataset. As can be seen, the IH33 is shifted right for PCS individuals compared to healthy controls. This shift is indicative of an increase in time between IH33 intervals, and hypothetically may be related to a reduction or slowing of efferent input. Therefore, the calculated feature comprised the total percentage of the interval histogram with bin values of more than 90 ms.

**Figure 3:**
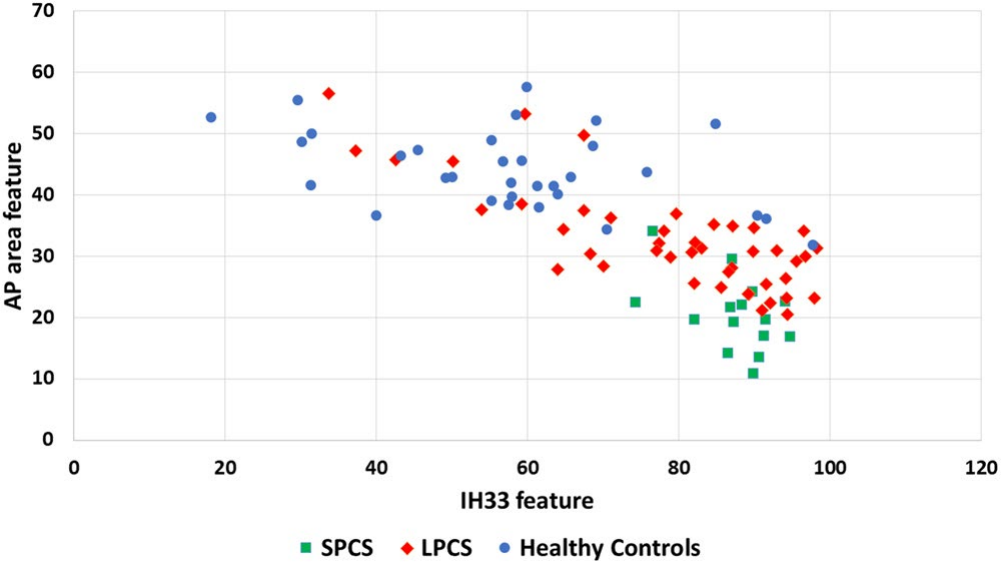
Scatter plot of healthy controls (n = 33) versus PCS group (n = 59) using AP-area and IH33 features. The two subbroups of short- and long-term PCS (SPCS, n = 15 and LPCS, n = 44) are shown with different colors and markers.

Figure 3 shows the scatter plot of PCS individuals (n = 59) versus healthy controls (n = 33) using the same two PCS features (AP area and IH33) as in^26^. Similar to our previous study, for this figure we show the clusters of PCS individuals with short-term injury (concussion <3 months prior to recording), called SPCS, and PCS with long-term injury (concussion >3 months prior to recording), called LPCS, versus controls. Congruent to the previous results, the new PCS individuals (both SPCS and LPCS) were found within the PCS cluster. Moreover, the new SPCS individuals were classified with the SPCS cluster, which was always more distal from controls compared to the LPCS.

When applying the same classifier trained with the previous study’s^26^ data (38 PCS and 33 healthy participants) to the new 21 PCS data (14 LPCS and 7 SPCS) using the same AP area and IH33 features a blind test accuracy of 95% was achieved. This was even higher than the 84% test accuracy reported previously^26^. This increase in accuracy was likely due to the increased ratio of SPCS to LPCS in the new population.

### EVestG Depression feature

**Figure 4:**
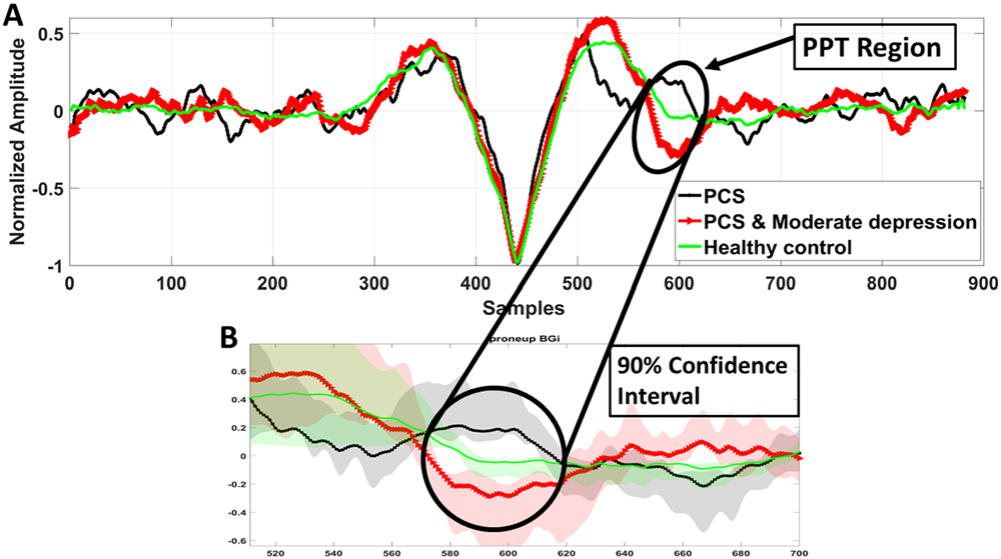
(**A**) Average FP curves during static segment of PCS individuals with no depression (MADRS score: 0–6, n = 18) and PCS with moderate/severe depression (MADRS score > 20, n = 14) in comparison to that of the healthy controls (n = 33). The marked circles/arrows show significant (P < 0.1) difference in the PPT region (Samples = 571:619) between the three groups.

Within a population of Major Depressive Disorder (MDD) patients, Lithgow *et al*.^32^ showed using 3 features one of which was the post potential trough (PPT) region of the FP signal of the EVestG (Fig. 1D) could separate between MDD and healthy controls with high leave-on-out-cross-validated accuracy (~87%). The best classifier feature between controls and MDD (feature ROC = 0.75) was found to be the left side PPT region of the FP^39,40^. Herein, within our PCS population, we compared the average FP of non-depressed PCS and depressed PCS groups using this PPT area feature extracted from static segments (Fig. 1D). The PPT area is defined as the area bounded by samples (571:619) based on the average healthy control FP curve (Fig. 1D). As shown in Fig. 4, this area was found marginally significant (p = 0.06) different between PCS with no depression (n = 18) and PCS with moderate/severe depression (n = 14) and also when comparing each of these two groups with the healthy control group (p = 0.08). On the other hand, no significant difference was found between these two groups (PCS with no depression and PCS with moderate/severe depression) and PCS with mild depression.

**Figure 5:**
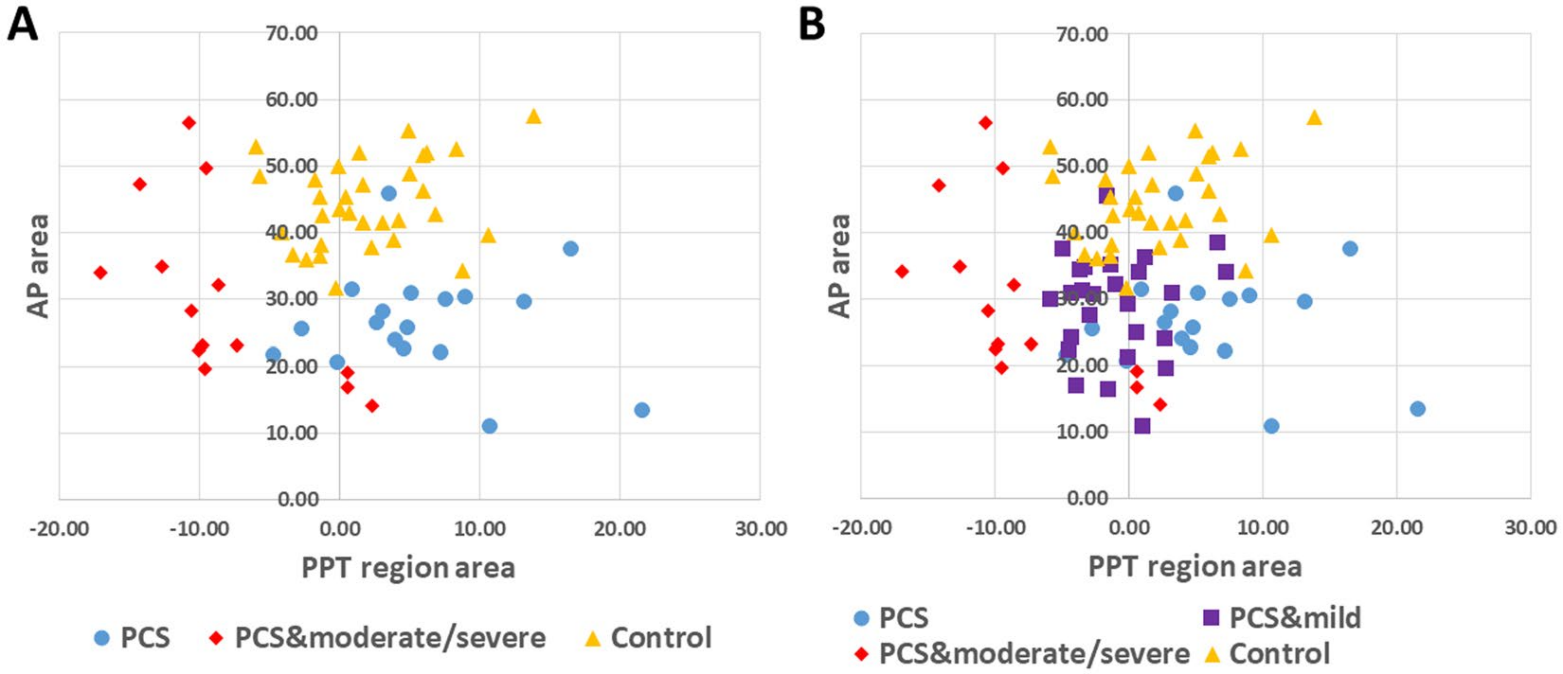
Combination of the PCS feature (AP-area) and the depression feature (PPT region) for separating the groups of (**A**) PCS with no depression (MADRS < 6, n = 18), PCS with moderate/severe depression (MADRS > 20, n = 14) and healthy control (n = 33). (**B**) When PCS with mild depression (MADRS: 7–19, n = 27) was included.

**Figure 6:**
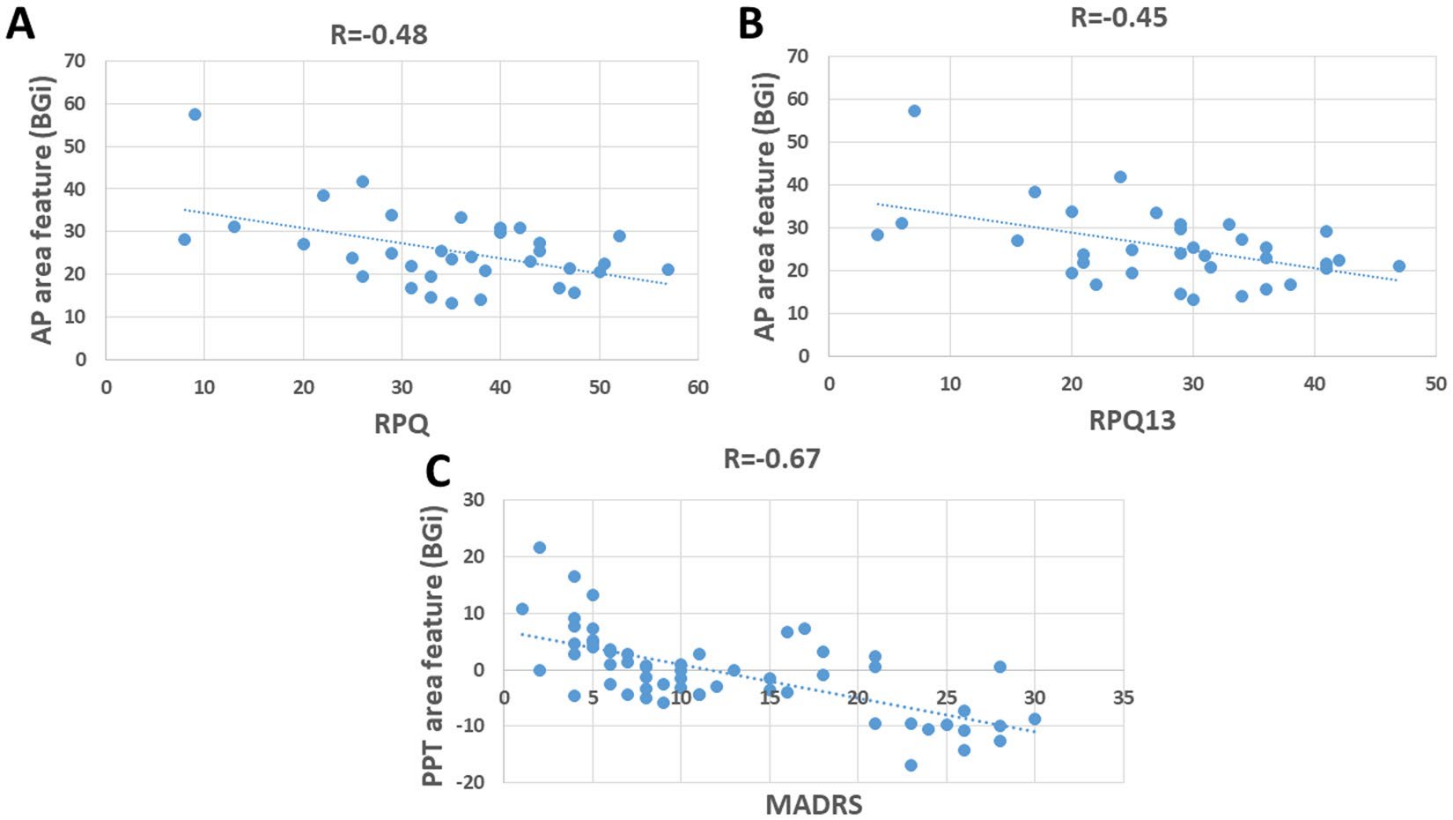
(**A**) The correlation (R = −0.48) between RPQ total and the PCS specific feature (AP-area). (**B**) The correlation (R = −0.45) between RPQ13 and the PCS specific feature (AP-area). (**C**) Correlation (R = 0.67) between MADRS score and the depression specific feature (PPT area).

Figure 5 shows the scatter plot for the three different PCS depression severity groups using a combination of AP-area and PPT features. The AP-area ideally classifies PCS, whilst the PPT region area classifies depression. However, these two features extracted from the PCS individuals were significantly correlated (R = −0.28, P = 0.03). On the other hand, no significant (R = 0.05, P = 0.7) correlation was found between these two features when the healthy control population was included (Table 3). As can be seen in Fig. (5A,B), the PPT feature provided good separation between the three PCS depression severity groups. Moreover, a significant correlation was found between the EVestG depression feature (PPT area) and the MADRS score (R = 0.67, P < 0.01), while no significant correlation was found between the EVestG PCS feature and the MADRS score (R = 0.20, P = 0.12). Figure 6C shows an example of the association between the PPT area and the MADRS score. As can be seen, the larger the PPT area (trough area becomes more negative compared to healthy control Fig. 4), the higher the MADRS score which in turn implies an increase in depression level.

**Table 3:** Calculated correlation between the neurophysiological assessments scores including RPQ and MADRS versus the PCS and the depression-specific features (AP-area and PPT area respectively) (n = 26).

	AP-area	PPT area	IH33
RPQ	−0.48*	0.07	0.21
RPQ3	−0.22	0.08	0.14
RPQ13	−0.45*	0.12	0.24
MADRS score	0.16	0.67*	0.20
PPT area	−0.28* (without healthy control) 0.05 (including healthy control)	—	0.06 (without healthy control) −0.11 (including healthy control)
AP-area	—	—	−0.57*
*p < 0.05			

The AP-area was extracted from the average static segment (BGi) while sitting upright, while the PPT area was extracted from the static segment while sitting in a supine position. All the correlation were calculated without including control data except if it is mentioned.

Using linear discriminant analysis (LDA) incorporating a leave-one-out routine, we calculated the accuracy of separating each of the three PCS depression groups from healthy controls. The features used for classification were: the AP-area, IH33 plot and the PPT area. Table 4 shows the resultant accuracy using each feature alone, while Table 5 shows the resultant accuracy of the combinations of two and three features. As expected, the best combination of the features resulted in higher accuracies, namely, 100% for separating PCS with no depression from healthy controls, and ~94% for separating PCS with moderate/severe depression from healthy controls. Using LDA with a leave-one-out routine, we also calculated the 4 group (3 PCS and Healthy) classification accuracy as well as 3-groups classification (PCS with mild depression subgroup excluded) accuracy as 74% and 81.5%, respectively (Table 6).

**Table 4:** LDA classification accuracies using a leave-one-out routine for three features: AP-area, PPT area and IH33.

	AP-area	PPT area	IH33
PCS (no depression) VRS. Control	92%	62%	86%
PCS & mild depression Vrs. Control	88%	61.7%	81.7%
PCS & moderate/Severe depression Vrs. Control	83%	85%	72%

Testing accuracy of each feature for separating each of the three PCS groups (PCS (n = 18), PCS & mild depression (n = 27) and PCS & moderate/severe depression (n = 14)) from healthy controls (n = 33).

**Table 5:** LDA classification accuracies using a leave-one-out routine for three features: AP-area, PPT area and IH33.

	AP-area & IH33	AP-area & PPT area	AP area, PPT area and IH33
PCS (no depression) vrs. Control	100%	94%	94%
PCS & mild depression Vrs. Control	90%	83.3%	90%
PCS & moderate/SEVERE depression Vrs. Control	83%	93.6%	89.4%

Testing accuracy of two and three features combination for separating each of the three PCS groups (PCS & no depression (n = 18), PCS & mild depression (n = 27) and PCS & moderate/severe depression (n = 14)) from healthy controls (n = 33).

**Table 6:** The calculated accuracy of LDA with leave-one-out classification using 3 features for separating PCS with no depression versus PCS & mild depression versus PCS & moderate/severe depression versus healthy control.

	Accuracy	#Features	Features
4 groups classification	73.9 %	3	AP-area, PPT area and IH33
3 groups classification*	81.5%	3	AP-area, PPT area and IH33
3 groups classification*	80%	2	AP-area and PPT area
3 groups classification*	75.4%	2	PPT area and IH33
3 groups classification*	64.6%	2	AP-area and IH33

*Without including PCS & mild depression subgroup. The 3 group classification accuracy was calculated as well using 3 and 2 features when the PCS & mild depression subgroup was excluded.

We also investigated the correlation between the EVestG features (AP-area and IH33) and the RPQ scores (Table 3). It should be noted that we had RPQ scores for only 26 study participants; the rest were recorded in the first study wherein RPQ was not included in that study’s assessment. The AP-area showed significant correlation with RPQ (R = −0.48, p = 0.003) and RPQ13 (R = −0.45, p = 0.004) but not with RPQ3 (R = −0.22, p = 0.20). No significant correlation was found between IH33 and the RPQ scores (Table 3). Figure 6A,B show examples of the association between the AP-area versus RPQ (Fig. 6A) and RPQ13 (Fig. 6B), respectively. As can be seen, the narrower the AP-area, the higher was the RPQ/RPQ13 score; that implies a decrease in AP-area represents an increase in PCS symptoms severity.

## Discussion

Brain injury can affect how neurons process and transmit information between cells. While affected cells usually recover after an injury, some may degenerate and die^41^. The neurological changes are identified acutely in the first week post-injury and for individuals with persistent PCS even much later^3^.

The results of this study indicate that the classifier accuracy based on the two PCS-specific features only produce a high accuracy in classifying PCS from healthy control. However, the performance of this classifier reduces when depression is comorbid. Consequently, we improved the classifier by increasing its dimension from two to three with the addition of an EVestG derived depression feature. This resulted in a better accuracy (from 64% to 81%) for separating the PCS with depression group from the healthy control group.

In the previous study^26^, EVestG was shown to have potentials as a reliable diagnostic assistive tool for PCS and have the ability to provide a measure for recovery from PCS. In addition, the EVestG signal analysis was shown to classify both short- and long-term PCS (SPCS and LPCS) from healthy controls and also from each other with high accuracy. Herein, we tested our previously developed classifiers on a new set of recorded data. The calculated accuracy increased from 84% to 95% when blind tested with new data. This accuracy improvement is likely due to a larger percentage of SPCS rather than LPCS subjects who are more likely to be correctly classified. This indicates that our developed classifier for separating PCS and healthy control is likely valid and reliable. It is noteworthy too that of the 21 new data only one was misclassified. As shown in Fig. 3, using the same calculated PCS features as in^26^, we still see two clear clusters of SPCS and LPCS participants. The responses of the AP-area and IH33 features for LPCS are closer to healthy controls compared to SPCS (Fig. 2C,D).

To diagnose PCS, it is imperative for clinicians to systematically evaluate and eliminate the possible contribution of co-morbidities and/or socio-psychological factors that may cause or maintain self-reported symptoms after a mTBI. Depression and anxiety are considered common clinical conditions that often occur after a mTBI which may be due to chronic pain such as headaches and neck pain that might be caused by whiplash^17^.

Depression is considered as one of the most persistent and confounding differential diagnoses for the PCS^10,42^. It has been reported that PCS can become more severe when comorbid with depression^43^. In this study, the average time between the mTBI event and the EVestG recording was 3.5 ± 5.3 yrs for the “PCS & moderate/severe depression”, 2.1 ± 3.4 yrs for the “PCS & mild depression” and 0.8 ± 0.6 yrs for the “PCS with no depression” (Table 2). This indicates that depression score may increase with time since the injury.

Given that EVestG technology is also sensitive to depression and perhaps mood disorders^32,37,44^ and has been used to measure the symptomology of depression^32,37^, we investigated the effect of the comorbid depression on PCS and whether that can be teased out using the EVestG technology. The answer to this question can be seen in Fig. 5B. The three subgroups of PCS with depression were cluster-wise clearly identifiable along the X-axis (the depression (PPT) feature). Of particular note is having the PCS with moderate/severe depression clustered more distal compared to both PCS with mild depression or PCS without depression (Fig. 5A,B). The PCS feature (AP-area), as presented in this figure, showed a significant correlation (R = −0.28, p = 0.03) with the depression. As a result, the AP-area tends to become slightly wider as depression severity increases (Fig. 4A) potentially confounding the PCS detection.

In our previous study on PCS population^26^, the neurophysiological changes that may take place post injury and could lead to a narrowing of the AP-area were hypothesized. In summary, the narrowing of the AP-area was argued to be due to an excessive of efflux and influx of sodium potassium ions through the membrane, and this change has been argued to be due to the accumulation of calcium ions (Ca^+2^) inside the injured nerves^26^. On the other hand, the PPT region of the FP is more likely generated as a combination of peripheral and brainstem response activity^45–47^, and likely corresponds to the repolarization mechanism. This is based on the hypothesis that the PPT region is comparable with the N2 component of the acoustic compound action potentials (Fig. 1D). Traditionally, the acoustic N2 peak was thought to be generated in the brainstem and this view was primarily based on the observation that when the cochlear nucleus (CN) was removed, or the central end of the cochlear nerve was sectioned, the N2 peak was abolished^46–49^. Later, it was shown also that sectioning the cochlear nerve produced only a reduction in the N2 peak amplitude^45^. Considering the fact that Vestibular^50^ and the acoustic compound action potentials have similar characteristic shape and both are comparable with the extracted vestibular FP of the recorded signals. We believe that the PPT region of the vestibular FP is also a combination of peripheral and brainstem response activity. Lastly, in a study on depressed population^32^, it was shown that the average repolarization mechanism in depression was slower than in healthy controls.

Herein, when comparing PCS with no depression and PCS with moderate/severe depression, the repolarization mechanism and in particular the P1 and N2 (see Fig. 1D) peaks appear to occur with longer latencies (Fig. 4). We hypothesize the result is a significant FP waveform difference in the PPT region. The generation of P1 and N2 peaks (see Fig. 1D) of the FP corresponds to the influx of the potassium ions (K^+^)^26,45^. It was argued in^26^, that PCS individuals are characterized as having increased potassium ions (K^+^) current influx, and this was one reason behind the narrowing of the AP-area for PCS individuals. This repolarization mechanism appears to continue being faster in the PPT region (P1 and N2 peaks). Herein, we further hypothesize this increased flux can also help explain why the generation of the P1 peak is faster for PCS with no depression compared to healthy controls.

When there is comorbid depression associated with PCS, the depression appears to slow this mechanism, P1’s and N2’s latencies increase to become more control like and depression like, respectively. Though not significant (P = 0.2), the AP-area of the PCS with moderate/severe depression (red line) is wider than for PCS with no depression (Fig. 4A, black line); i.e. the mechanism of the efflux and influx of sodium (Na^+^) potassium (K^+^) ions may also slow with depression. This decrease continues to be observed in the PCS population with moderate/severe depression group in the PPT region as the potassium ions (K^+^) influx has a slowed depression component thus, we hypothesize it to be acting in opposition to the faster PCS component and potentially confounding the PCS measures.

From the previous^26,28^ and current studies, the AP-area is considered as a robust feature for separating PCS from healthy control. However, it is not as true when it comes to the classification of PCS with comorbid depression versus healthy controls (Tables 4 and 5). Comorbidity of PCS and depression can result in a slightly wider AP-area closer to AP-area of healthy individuals. The presence of depression resulted in a decrease in classification accuracy from 100% in PCS with no depression to 83% for PCS with moderate/severe depression (Table 5). By adding the third (depression) feature, the PPT area, to the previous features, the calculated accuracy improved to 89% for classifying PCS with moderate/severe depression from healthy controls (Table 5). The AP and PPT features have the least correlation between them (Table 3) and interestingly, the use of these two features alone improved this classification accuracy to 93% (Table 5). Therefore, the presence of depression in a person with a history of brain injury can make it potentially more challenging to diagnose persistent PCS, given the interplay of symptoms. However, the overall results show that the combination of the AP-area, IH33 and PPT area feature resulted in the best accuracy for four and three (excluding PCS with mild depression subgroup) way classifications (Table 6). Thus, using the PCS (AP-area and IH33) and depression (PPT area) specific features we may be able to assist in the detection of someone having symptoms resulted from a head injury, depression or both.

To test the association between the AP-area extracted feature and the severity of the PCS, we calculated the correlation between the AP-area and the RPQ scores. The resultant correlations were significant between the AP-area and RPQ13 (R = −0.45, p = p = 0.004) but not RPQ3 (R = −0.22, p = 0.20). This indicates that AP-area is more likely associated with the symptoms which are common to during later stages of the injury^11^ as characterized in the RPQ13 score and less so with RPQ3 and the symptoms characterizing the early stage of the injury^11^.

In this study, we showed that when depression and PCS are comorbid in a PCS group, the EVestG features could be used to detect both conditions with two different and relatively independent neurophysiological mechanisms that can be applied simultaneously.

The main limitation of this study is its relatively small sample size. The main finding of this study is that EVestG has the potential to and appears is a reliable tool for assisting in the diagnosis of PCS with and without the comorbidity of PCS and depression.

## Methodology

All the methods and experimental procedures of this study were approved by the University of Manitoba Biomedical Research Ethics Board, and all the participants signed an informed consent prior to the experiment. All experimental procedures were performed in accordance with the protocol approved by the Biomedical Research Ethics Board and its regulations.

### EVestG recording

A typical EVestG signals recording is conducted on a hydraulic chair inside an electromagnetically shielded and sound attenuated (>30 dB) chamber with eye closed and head supported to minimize the muscle artifacts. The placement of the electrodes includes two electrodes resting close to the tympanic membrane of each ear (Fig. 1B), reference electrodes on each ipsilateral earlobe, and a ground electrode on the forehead. The recordings were made whilst the chair was static and moving^29^.

### Neuropsychological assessments

Besides the EVestG assessment, participants also completed two neuropsychological assessments: MADRS^39,40^ and RPQ^11,51^.

MADRS is a commonly used instrument in depression research to measure the severity of depression^40^. It contains 10 diagnostic questions with a total score of 60. Herein, we used MADRS to measure the depression severity among our PCS population. Based on the MADRS score, the PCS population was divided into three subgroups: (1) PCS with no depression (MADRS score < 6, n = 18), (2) PCS with mild depression (7 < MADRS score <19, n = 27), and (3) PCS with moderate/severe depression (MADRS score >20, n = 14).

The RPQ score was used for calculating the severity of the PCS^11,51^. This questionnaire consists of 16 post-concussion symptoms, and for each symptom, there is a score from 0 to 4 as an indication of the severity of that specific symptom. In this study, we divided the RPQ score into two sub-scores: (1) RPQ-3, which is the score of the first three symptoms of RPQ (headaches, dizziness and nausea) which are particularly common in the early stage post-injury^11^ and (2) RPQ-13, the score of the other thirteen symptoms that are mostly cognitive and emotional symptoms and particularly common as later PCS symptoms^11^.

## Data Availability

The data that support the findings of this study are available from Neural Diagnostics Pty. Ltd. but restrictions apply to the availability of these data, which were used under license for the current study, and so are not publicly available. Data are however available from the authors upon reasonable request and with permission of Neural Diagnostics Pty. Ltd.
